# Structural Path Analysis of Fossil Fuel Based CO_2_ Emissions: A Case Study for China

**DOI:** 10.1371/journal.pone.0135727

**Published:** 2015-09-02

**Authors:** Zhiyong Yang, Wenjie Dong, Jinfeng Xiu, Rufeng Dai, Jieming Chou

**Affiliations:** 1 State Key Laboratory of Earth Surface Processes and Resource Ecology, Beijing Normal University, Beijing, People’s Republic of China; 2 Future Earth Research Institute, Beijing Normal University Zhuhai, Zhuhai, Guangdong Province, People’s Republic of China; Nanjing University, CHINA

## Abstract

Environmentally extended input-output analysis (EEIOA) has long been used to quantify global and regional environmental impacts and to clarify emission transfers. Structural path analysis (SPA), a technique based on EEIOA, is especially useful for measuring significant flows in this environmental-economic system. This paper constructs an imports-adjusted single-region input-output (SRIO) model considering only domestic final use elements, and it uses the SPA technique to highlight crucial routes along the production chain in both final use and sectoral perspectives. The results indicate that future mitigation policies on household consumption should change direct energy use structures in rural areas, cut unreasonable demand for power and chemical products, and focus on urban areas due to their consistently higher magnitudes than rural areas in the structural routes. Impacts originating from government spending should be tackled by managing onsite energy use in 3 major service sectors and promoting cleaner fuels and energy-saving techniques in the transport sector. Policies on investment should concentrate on sectoral interrelationships along the production chain by setting up standards to regulate upstream industries, especially for the services, construction and equipment manufacturing sectors, which have high demand pulling effects. Apart from the similar methods above, mitigating policies in exports should also consider improving embodied technology and quality in manufactured products to achieve sustainable development. Additionally, detailed sectoral results in the coal extraction industry highlight the onsite energy use management in large domestic companies, emphasize energy structure rearrangement, and indicate resources and energy safety issues. Conclusions based on the construction and public administration sectors reveal that future mitigation in secondary and tertiary industries should be combined with upstream emission intensive industries in a systematic viewpoint to achieve sustainable development. Overall, SPA is a useful tool in empirical studies, and it can be used to analyze national environmental impacts and guide future mitigation policies.

## Introduction

According to recent studies, the concentration of carbon dioxide (CO_2_) in the atmosphere has increased from approximately 277 parts per million (ppm) in 1750 [1] to 392.52 ppm in 2012 [2], and the average concentration exceeds 400 ppm for the first time in May 2013 [3]. Most of this growth since the industrial era can be ascribed to anthropogenic greenhouse gas (GHG) emissions, especially CO_2_ from fossil fuel combustion and cement production. These CO_2_ emissions have been 8.6 ± 0.4 GtCyr^-1^ on average during the past decade (2003–2012), three times the emission rates of the 1960s [4]. However, emission growth trends vary by nation and region. Developed countries (e.g., the US and western European countries) always show moderate CO_2_ growth, whereas developing countries (e.g., China) have increased their emission significantly in past decades [5]. Moreover, the magnitudes of CO_2_ emissions in developed countries have far surpassed those in developing countries since 1750, but in recent years, this trend has reversed [5]. All of these phenomena are highly correlated with the unprecedented expansion (more than 50%) of global energy demand since 1990, and in particular correlated with the rapid demand growth in non-Organization for Economic Cooperation and Development (non-OECD) countries since 2000 when China alone accounts for more than half of the increase [6]. Around 2011, China becomes the top CO_2_ emitter from both producer and consumer perspectives [7]. At the same time, most cities in North China are experiencing severe air pollution—annual particulate-matter (PM_10_) levels are 5–7 times the World Health Organization (WHO) guideline level [6], and annual sulfur dioxide (SO_2_) levels are triple the WHO 24-hour guideline level [8]. Together with global warming, these issues have caused growing public concerns, pushing fossil fuels (especially coal) to the top of the domestic political agenda, such as the five major actions on energy policy called for by Chinese President Jinping Xi in June 2014 [9]. Recently, China has declared a goal of reaching peak emissions before 2030, which indicates a sharp decrease in coal consumption and soaring demand for renewables [10]. Therefore, this work focuses on CO_2_ emissions from fossil fuels in China and tries to derive sound national and sectoral policies for future mitigation.

Often, input-output analysis (IOA) is used to analyze an economy in a structural way, considering both intermediate and final use. EEIOA, as an extended IOA, is a methodology that combines IOA with environmental externalities. Both single-region input-output (SRIO) and multi-region input-output (MRIO) versions of EEIOA have been implemented, concentrating on embodied emissions in international trade [11–12], emission responsibilities in producer, consumer and shared perspectives [13–19] and other studies about water footprint and biodiversity [20–21]. Another significant feature of EEIOA is its ability to display intricate sectoral inter-relationships along the production chain. The technique to excavate these linkages using EEIOA is called structural path analysis (SPA), which is often used in economics and regional science to analyze flows of energy, carbon, water and other physical quantities through industrial networks [22, 23–26]. Moreover, SPA can be combined with structural decomposition analysis (SDA) to do deeper analysis, such as structural path decomposition (SPD) [27–28]. Currently, most CO_2_ emission studies for China focus on major driving forces by using the index decomposition method [29–30] or the structural decomposition method [31–34]. Relevant studies also analyze embodied emissions in bilateral trade [35–36] and assign emission responsibilities in different perspectives [37]. However, few have focused on emission interactions between different sectors along the production chains, such as Huang et al. [23] and Lenzen et al. [14]. Therefore, in this paper, we set up the first quantitative study for China using the SPA method to extract and analyze CO_2_ emissions along the production chains from both sectoral and final use perspectives based on imports-adjusted national input-output tables.

The aim of this article is to understand the emission structures and the interdependence of sectors along the production chain and to derive sound policy for future mitigation. The remainder of this paper is arranged as follows: Section 2 describes the methods and materials used; Section 3 presents empirical studies with two different perspectives and discusses relevant policies; and Section 4 concludes the work and summarizes the implications.

## Materials and Methods

### Input-Output Analysis (IOA) and Import Assumptions

Based on the 2007 input-output (IO) table for China, a model is constructed to describe the total output of an economy:

X=AX+C+I+G+EX−IM+ERR (1)

where X is the total output, A is the direct requirement matrix, C is household consumption, I is investment, G is government spending, EX is exports, IM is imports, and ERR is the error item that is included for balance and is provided in the IO table. Solving this Eq (1) for X gives:

$$X={\left(I-A\right)}^{-1}*\left(C+I+G+EX-IM+ERR\right)=L*\left(C+I+G+EX-IM+ERR\right)$$ (2)

where L represents the Leontief inverse matrix and X is the same as that given in (1). Combining X with the environmental intensity row vector F, which represents CO_2_ emissions per unit of sector output in this study, total CO_2_ emissions (EM) can be formulated as follows:

EM=F*X=F*L*(C+I+G+EX−IM+ERR) (3)

This is the so-called environmentally extended input-output model (EEIOA).

There is no doubt that an MRIO model is more appropriate than an SRIO model for estimating the actual carbon emissions embodied in trade because differences in environmental intensities and production chain structures in various nations are built into one model [38–39]. Using MRIO with SPA makes it possible to simultaneously consider the global production chain and calculate detailed importing emissions originating from domestic final use [24–25, 38]. Nevertheless, construction of an integrated MRIO model is difficult due to data availability, time, various assumptions and harmonization work. An SRIO model, in contrast, is often self-contained, and the relevant data can be readily collected. Using competitive SRIO (as discussed below) with SPA [14, 23], it is also possible to consider the global production chain, although detailed importing sources are unavailable. In general, no universally superior model exists, there are only models that are more or less appropriate for specific purposes [39–41]. If the purpose is to analyze the incurred impacts from domestic produtive activities (namely C, I, G and EX excluding importing elements) and the sectoral inter-relationship along the domestic production chain, an SRIO model may be more appropriate due to data availability and computational resources. It is also reasonable to use an SRIO model because real transactions between industries usually can be assumed to happen within a nation, although incurred emissions might transcend the boundaries. Due to these facts, an adjusted SRIO model with 56 sectors for 2007 Chinese economy is used in this paper.

The treatment of imports in SRIO studies is different. Miller and Blair [42] draw a distinction between imports of goods that are also domestically produced (competitive imports) and those for which there is no domestic source (noncompetitive imports). This difference leads to 2 types of imports assumptions in SRIO: competitive and non-competitive [43]. Most OECD countries use the non-competitive assumption, but China and United States use the competitive one. Based on this assumption, China merely publishes the competitive IO tables. The “direct requirement matrix” in competitive IO table is also called the “regional technical coefficients matrix”, and it includes intermediate use imports and can describe the real technology interrelationship of regional industries [42]. In contrast, the assumption behind direct requirement matrix in non-competitive IO table cannot accurately describe the technology of regional firms; rather, it describes the way in which local firms use local inputs [42]. Furthermore, the competitive imports assumption in SRIO implies that the environmental intensities and supply chain structures in foreign nations are the same as those in the country being analyzed [38–39]. This is often called “Domestic Technology Assumption” (DTA) [44], the “import assumption” [45–46] or the “single region assumption” [47]. Although this might cause errors in emissions embodied in imports [24, 44–45, 48–49], it is still supposed to be a balanced result considering time horizon, type of data, cost and work effort, detail and comprehensiveness [50]. Because our purpose is to grasp the real interrelationship between industries along the production chain, we use the “regional technical coefficients” and the corresponding competitive imports assumption.

SRIO studies for China often treat imports as goods or services produced abroad, and many of them fail to distinguish between final uses that come from domestically produced (C_1_, I_1_, G_1,_ EX_1_) and imported (C_2_, I_2_, G_2,_ EX_2_) sources [32–33], or they may simply assume that each economic sector and final demand category uses imports in the same proportions [36, 51]. Because our purpose is to see the incurred impacts from domestic final use, imports should be subtracted. Therefore, some elements in EX and IM are first eliminated (such as EX_2_), then 2007 Custom statistics in China are used to subtract the importing elements (C_2_, I_2_, G_2_) from the remaining C, I and G. After removing all these importing elements from final use and imports (IM), we derive Eq 4:

$$EM=F*X=F*L*\left(C_{1}+I_{1}+G_{1}+{EX}_{1}-{IM}_{1}+ERR\right)$$ (4)

where IM_1_ is imports for intermediate use. This modified IO table and Eq (4) can display actual domestic final use and real inter-dependence of industries simultaneously.

### Structural path analysis (SPA)

The Leontief inverse can be expanded using a power series approximation [52], and if we treat (C_1_+I_1_+G_1_+EX_1_-IM_1_+ERR) as a single vector Z, then

$$EM=F{\left(I-A\right)}^{-1}\;Z=FIZ+FAZ+FA^{2}Z+FA^{3}Z+\cdots \cdots$$ (5)

where FA^t^Z represents the impact from t^th^ production tier. For example, if Z represents a demand on the production of a plane, then FIZ is the direct manufacturer pollution from the production process. To produce this plane, inputs AZ from other industries are required, and FAZ of pollution is emitted. In turn, these industries require inputs of A^2^Z and emit FA^2^Z of pollutant. This process continues through the infinite expansion of the power series. Finally, the whole picture of pollution derived from the production of the plane is drawn, and the shape is similar to a “tree data structure” in computer science.

Similar to the classical “80–20 Rule”, top several tiers contribute most of the influences, and within each tier, a limited number of nodes and routes are of great importance [14, 53]. The “nodes” are influences (e.g., CO_2_ emissions) that are used to satisfy final uses downstream, and “routes” are linear demonstrations of these influences upstream in the production chain. If there are N sectors in an economy and the tier number is M, the total number of nodes in that tier will be N^M^, which can be innumerable in some circumstances. However, we only need to extract large nodes and routes within the top several tiers in order to grasp the whole picture. The basic technique used to extract these important nodes and routes along the production chain is referred to as Structural Path Analysis (SPA) [54]. Combined SPA with the selected depth of tiers, a cut-off threshold can be implemented to screen nodes above a certain level, thus setting up the boundaries of extraction. This is also called the “pruning method” [24–26, 53, 55]. In this paper, sectors and routes are described using short English descriptors (see Table A in S1 File). For instance, “Power” and “Constr” represent the power sector and construction sector, respectively, and “Constr-Power” is the emission route originating from the power sector to satisfy downstream construction demands.

Table 1 reveals that the first 8 tiers contribute nearly 90% of the total emissions for each domestic final use item. Tier 0 contributes most in rural and urban consumption, and Tiers 1 and 2 contribute the largest shares in investment, government spending and exports. This indicates that, for consumption, most emissions are embodied in direct purchase. For exports, investment and government spending, the majority of emissions originate from upstream sectoral interactions in the first several tiers. Based on existing studies, the depth of the tier number selected lies between 5 and 10 [24–26, 53, 56–57] and the cut-off threshold can be displayed either in exact values [56–57] or in percentages, commonly ranging from 0.001% to 1% [14, 23–26, 53]. In this paper, the largest tier number is 8, and 0.001% is chosen as the cut-off threshold.

**Table 1:** Contribution of top 8 tiers to incurred emissions from each final use element (%).

Tier	Rural consumption	Urban consumption	Government Spending	Investment	Exports
0	39.79	28.45	7.76	3.62	9.58
1	14.61	17.25	19.93	24.59	16.77
2	11.75	13.99	18.11	19.62	17.15
3	9.30	11.07	14.71	15.50	14.92
4	7.09	8.43	11.25	11.36	11.73
5	5.20	6.20	8.33	7.95	8.74
6	3.72	4.44	6.00	5.47	6.32
7	2.62	3.13	4.25	3.74	4.49
Sum	94.09	92.95	90.35	91.85	89.70

Direct household emissions are included in tier 0 of the rural or urban consumption. And investment is the sum of fix capital formation and inventory change.

### Data preparation

Two types of data are needed: the IO table and the CO_2_ emission data by sector. The National Bureau of Statistics of China (NBS) publishes a series of detailed basic IO tables for the Chinese economy in 1992, 1997, 2002 and 2007. The 135-sector symmetric IO table (in producer price) for 2007 is selected from the INPUT-OUTPUT TABLES OF CHINA [58] and is aggregated into 56 sectors (see Table B in S1 File) to carry out the empirical study. This aggregation method aims to harmonize both the economic sectoral data and energy sectoral data, and at the same time reflect enough details satisfying the purpose of our research. For example, the 27 sectors (“96–107”) in 135-sector IO table are aggregated into 8 sectors (“39–46”) in 56-sector IO table (see Table B in S1 File) in order to match the “42” sector in energy statistics, as shown in Table C in S1 File.

General Administration of Customs (GAC) of China classifies the total merchandise trade into 19 sub-groups, including general trade, processing trade, inbound and outbound goods in bonded warehouses and storage of transit goods in bonded zones [59]. According to the definitions in INPUT-OUTPUT TABLES OF CHINA, EX and IM are total exports and imports, minus the processing trade values [58]. In order to achieve domestic final use elements, the value of goods imported and exported in bonded warehouses or zones is first separated from IM and EX (for merchandise trade sectors, 1–34), respectively, because these goods under bond administration are mainly preserved in the bonded warehouse or processed for export and will not enter domestic economic circulation [41]. The other three importing sub-groups (processing imported equipment, imported commodities and equipment as investment of foreign enterprises and imported equipment for export processing zones) are kept separate from the value of capital formation in sectors 1–34 because they are mainly used for investment purpose and are not generally used by households or government spending. The remaining imported goods in IM are separated into intermediate inputs and final use elements (excluding exports) using two methods. (1) For sectors 1–37, detailed merchandise imports in Harmonized System (HS) codes (version 2007) are selected from the China Customs Statistical Yearbook [59]. Combining these data with the mapping relationships (from sectors to HS codes) in Sheng [60] and the Broad Economic Categories version 4 [61], we can get the proportions of intermediate goods, capital goods and consumption goods in these 37 sectors (see Table D and Figure A in S1 File). Using these proportions, imported elements in capital goods and consumption goods are eliminated from C, I and G. (2) For sectors 38–56, imports are prorated according to values of intermediate and final use elements (excluding exports) in each IO sector, and those allocated to final use are subtracted from C, I and G. Finally, the modified IO table can display domestic final use and real production chains at the same time, which is different from both the non-competitive and competitive IO tables.

CO_2_ emissions data by sector are constructed using data from the Energy Statistics Yearbook [62]. Final energy consumption by industry sector in physical units and energy balance sheet in physical units [62] are combined and then transformed into energy units using net calorific values based on the GHG Protocol Tool for Energy Consumption in China (GPTFEC) [63]. This makes it possible to display sectoral energy consumption. Energy used for transformation, energy losses and non-energy uses are all considered by using the procedures in Peters et al. [64]. Finally, sectors are aggregated or separated to match those in the 56-sector IO table (see Table C in S1 File), and energy combusted is transformed to CO_2_ emissions using the reference method [65] based on the fraction of carbon oxidized and carbon content parameters in GPTFEC [63]. Total fossil fuel based CO_2_ emissions from both industrial and household direct use are calculated to be 6158.72 million metric tons (Mt). This is similar to 6112.42 Mt from the Carbon Dioxide Information Analysis Center (CDIAC) [66] and 6326.37 Mt from the U.S. Energy Information Administration [67], which testifies to the validity of this method.

## Results and Discussion

**Fig 1:**
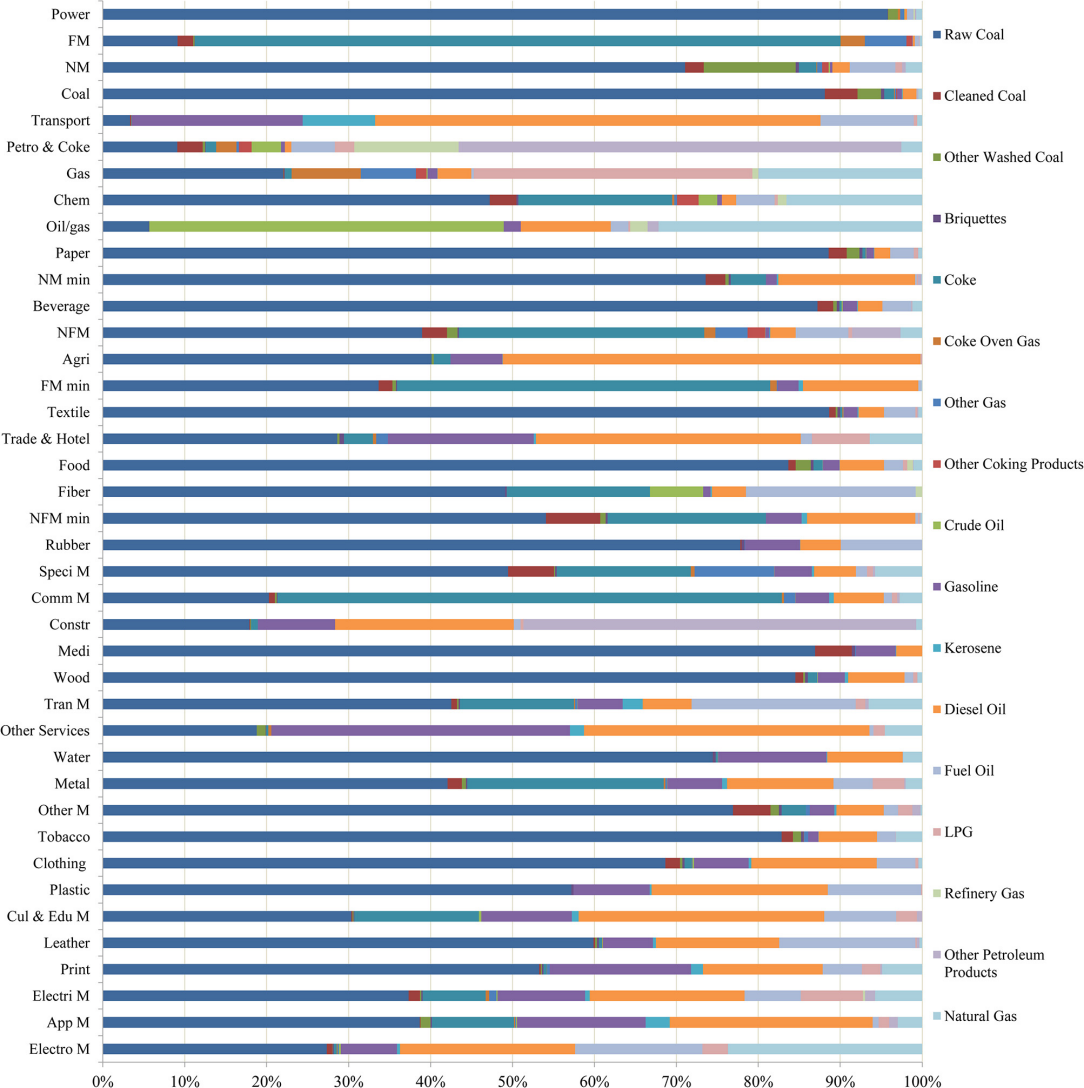
Sectoral emission intensity structures categorized in 17 final energy forms. The vertical axis shows 40 sectors in the descending order of their emission intensities from top to bottom. The horizontal axis shows the percentages of 17 final use energy forms for each sector. The transport sector includes Rail T, Land T, Water T, Air T, Pipe T, Public T, Storage and Post. Other services include all tertiary industries, except those in the transport sector (above) and the Trade & Hotel sector (namely Trade sector and Hotel sector).

**Fig 2:**
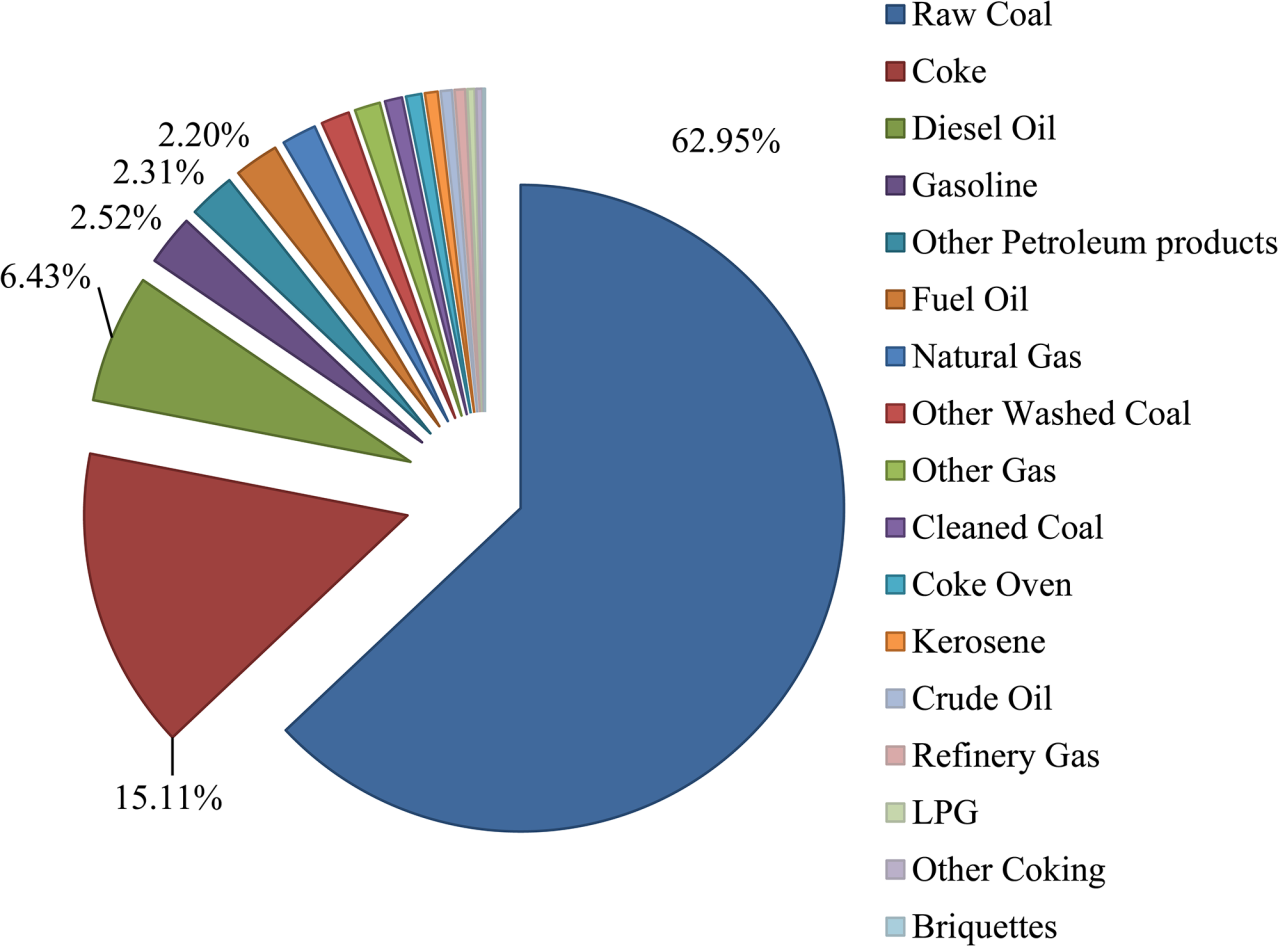
Percentages of CO_2_ emissions from different final energy forms in 2007 for China. Each sector in the pie chart represents the emission percentage of one type of final energy.

Considering sectoral emission intensities (see Fig 1), the largest one comes from power sector (951 g CO_2_/100 Yuan), which consumes almost four times as much as the FM sector. The following eight sectors have intensities greater than 50 g CO_2_/100 Yuan: NM, Coal, Transport, Petro, Coke, Gas, Chem and Oil/gas. Except for the transport sector, all top 10 sectors come from power generation or heavy industries. When we look into sectoral emission intensity structures considering various final energy use forms (as shown in Fig 1), there are great differences. For most sectors, raw coal, gasoline and diesel oil contribute the largest shares. For the top four sectors with the largest intensities, raw coal and coke contribute a great deal, whereas in the next five sectors (except Chem sector), petroleum, gas and relevant products dominate. From the industrial perspective, primary industry (Agri sector) relies greatly on raw coal and diesel oil. Secondary industry (37 sectors in total, see Table A in S1 File), however, varies greatly. For most industrial sectors, coke and raw coals contribute more than 40% sectoral emissions, whereas fuel oil, gasoline and diesel oil dominate the remaining share. For emissions in the Coke & Petro and Constr sectors, other petroleum products, refinery gas and oil products contribute the most. Sectoral emissions in Gas and Oil/gas highly depend on LPG, natural gas and crude oil due to their industrial characteristics. The Electon M sector is the only equipment manufacturing sector that has the lowest emission share in coal and highest share in natural gas. Tertiary industry can be summarized to three sectors due to our aggregation and disaggregation methods (see Tables B and C in S1 File): Transport sector, Hotel & Trade sector and Other services sector. Gasoline, kerosene, diesel oil and fuel oil dominate the emissions in transport sector, whereas raw coal, gasoline and diesel oil dominate the other two sectors. Taking all sectors together, the shares of CO_2_ from final energy consumed in 2007 are shown in Fig 2. Raw coal (62.95%) is the most important final energy form in China, coke (15.11%) is the second and is highly required in metal smelting and machine manufacturing, and diesel oil (6.43%) is third; it is particularly popular in Transport, Agri and Other service sectors.

To compare production-based emissions and incurred consumption-based emissions from final use sectors, we define the ratio of incurred emissions to production-based ones as “demand pulling indicator (DPI)”. DPI = 1 means sectoral emissions in production are equilibrium with those in consumption; DPI < 1 implies that this sector contributes more to satisfying other sectors’ demand than its own needs in the production chain; DPI > 1 indicates this sector depends on more emissions from other sectors in the production chain than its own production to satisfy its own demand. DPI is an indicator of sectoral roles (supplier or demander) in the production chain. Table 2 displays DPI values for 56 sectors. Power generation (Power), resource extraction (e.g., FM min, NM min, NFM min, Coal and Oil/gas), resource processing (e.g., FM, NM, Paper, Petro) and transport (e.g., Land T, Rail T and Public T) dominate the DPI indicators lower than 1, which means they are supporting other sectors in the production chain. Except for 3 sectors (NFM, Post and Water T) with DPI values around 1, all other 36 sectors have DPI values greater than 1. Equipment manufacturing sectors (e.g., Electro M, App M, Speci M) show significantly large DPI values ranging from 7.63 to 79.49. Construction (DPI = 33.20), Clothing (DPI = 26.48), Metal (DPI = 18.16) and various services sectors (e.g., Health, Edu, Real Est and Hotel) are also significant. It is clear that all of these 36 “demanding” sectors belong to downstream manufacturing industries or services and they are highly dependent on upstream transport, resources and power sectors. Downstream sectors with large DPI values can have a strong demand pulling effect on economy and emissions, and therefore, they should be treated seriously.

**Table 2:** Production-based emissions, consumption-based emissions and DPI indicators for 56 sectors in 2007.

Name	Prod	Cons	DPI	Name	Prod	Cons	DPI
Electro M	6.25	496.74	79.49	Trade	38.19	153.92	4.03
Electri M	6.95	352.82	50.75	Print	1.32	4.29	3.25
Cul & Edu M	1.07	49.02	45.79	Beverage	13.51	38.97	2.89
Constr	61.57	2044.02	33.20	Financial	13.97	31.53	2.26
App M	0.95	29.29	30.82	Fiber	5.15	10.19	1.98
Clothing	5.24	138.73	26.48	Air T	24.87	44.89	1.81
Health	7.97	181.44	22.75	Gas	6.99	10.13	1.45
Leather	2.64	58.92	22.34	Agri	113.86	162.86	1.43
Speci M	14.56	296.86	20.39	NFM	48.43	47.83	0.99
Metal	11.34	205.98	18.16	Water T	61.90	61.10	0.99
Admin	11.34	192.95	17.01	Post	98.08	95.30	0.97
Water	0.79	13.35	16.98	Public T	59.34	44.92	0.76
Edu	10.36	174.03	16.80	Chem	181.47	136.14	0.75
Tran M	23.93	397.11	16.60	Land T	94.94	40.67	0.43
Comm M	26.24	294.75	11.23	Rail T	35.01	13.81	0.39
Plastic	4.75	52.83	11.13	Coke	26.51	10.37	0.39
Wood	8.45	92.19	10.91	NM min	10.48	3.41	0.32
Recreation	2.54	26.23	10.33	Pipe T	3.82	1.23	0.32
Rubber	4.90	47.44	9.68	Paper	34.68	9.72	0.28
Other S	12.14	111.60	9.19	FM	1007.50	265.50	0.26
Rent	7.29	66.20	9.09	NM	344.59	85.54	0.25
Other M	6.39	46.99	7.36	Coal	103.73	14.10	0.14
Tobacco	2.23	15.67	7.04	Power	2994.92	396.66	0.13
Food	38.93	216.16	5.55	Petro	150.29	14.31	0.10
Textile	36.95	194.47	5.26	Storage	9.04	0.00	0.00
Real Est	10.59	53.40	5.04	Oil/gas	47.78	-14.97*	-0.31*
Hotel	19.62	94.36	4.81	NFM min	2.87	-2.49*	-0.87*
Medi	6.18	28.43	4.60	FM min	6.68	-12.02*	-1.80*

*These negative values in **Cons** and **DPI** can be ascribed to negative values inventory change.

Based upon the analyses above, we have drawn a basic picture of the sectoral emission patterns and their inter-relationship in the production chain. To further clarify the incurred CO_2_ emissions from a structural viewpoint and to develop sound mitigation policies, the SPA technique is implemented in both final use and sectoral perspectives in the following sections.

### Final use perspective

**Fig 3:**
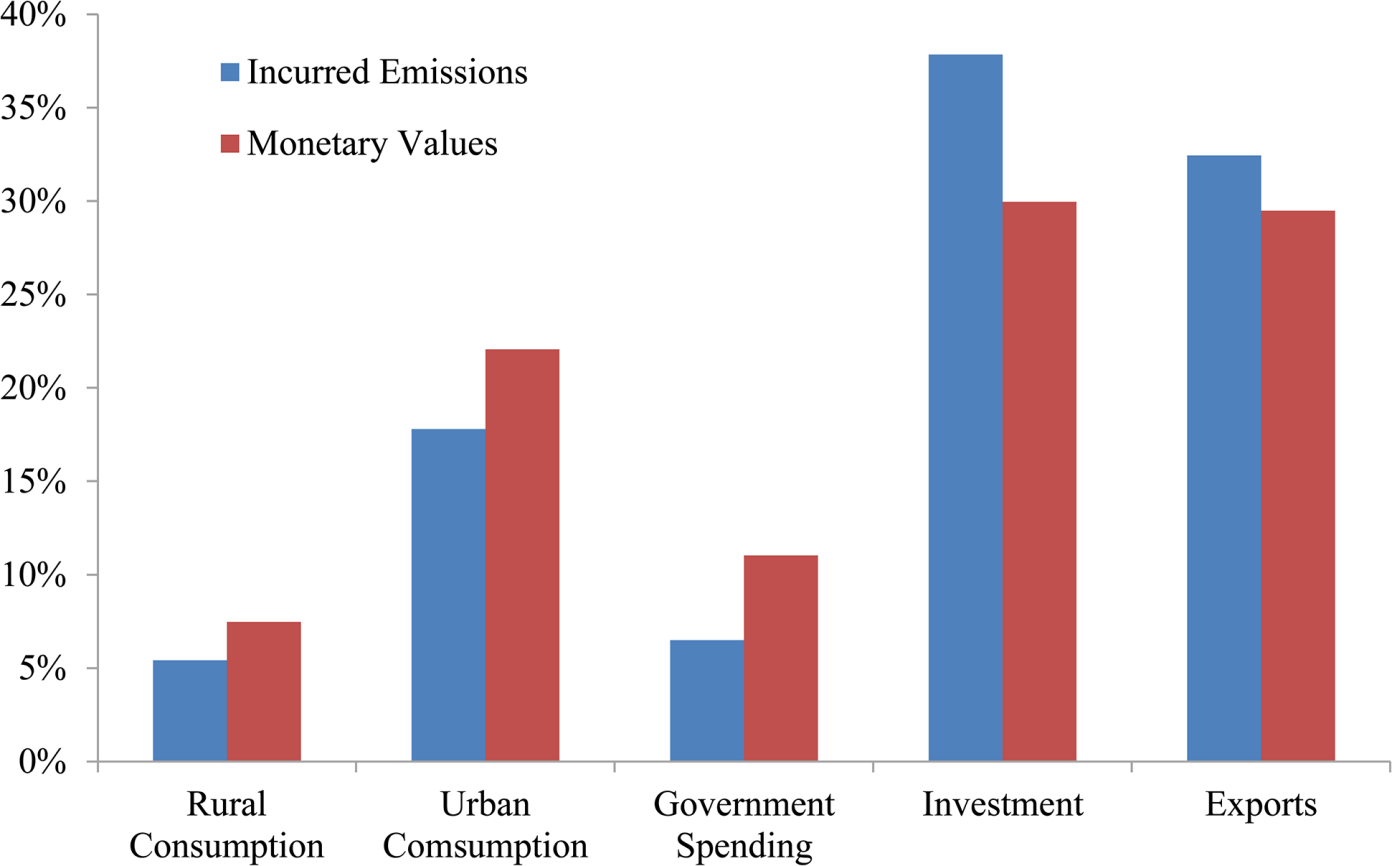
Shares of incurred emissions and monetary values from five domestic final use items. Red bar charts represent the percentages of monetary values, and blue bar charts represent ratios of incurred emissions. Investment is the sum of fix capital formation and inventory change. Direct household emissions are not included in the household emissions.

The bar chart in Fig 3 reveals monetary and incurred emission ratios of each domestic final use element. Similar to the monetary ratios, exports and investment dominate the total incurred emissions, but they are more emission intensive because they have higher ratios of incurred emissions than monetary values. This can be ascribed to large flows of investment in 2007 going to manufacturing sectors (32.41%), real estate (23.62%), transport, storage and post (10.31%) and public infrastructure and facilities (7.39%), which all have high DPI values [68]. Exports in 2007 mainly flow to equipment manufacturing (47%) and “textiles, light industrial products, rubber products, minerals and metallurgical products” (18%), which also have large DPI values [68]. Moreover, the low DPI values and large monetary amounts of services sectors (e.g., Admin, Land T and Public T) in government spending and household consumption can partly explain their lower emission ratios than monetary ones. Tables 3–7 demonstrate 25 top-ranking paths for CO_2_ originating from the household (rural and urban) consumption, investment, government spending and exports. All of these top paths are ranked in descending order due to their contributions to incurred emissions.

**Table 3:** Top 25 routes of CO_2_ emissions from Rural household Consumption.

Ranks	Data (Mt CO_2_)	Ratio (%)	Routes
0	126.16	23.35%	Direct energy use
1	48.27	8.93%	Power
2	17.31	3.20%	Power—Power
3	11.89	2.20%	Agri
4	6.21	1.15%	Power—Power—Power
5	5.26	0.97%	Post
6	4.55	0.84%	Agri—Power
7	3.80	0.70%	Edu—Power
8	3.78	0.70%	Food
9	3.38	0.63%	Food—Power
10	3.31	0.61%	Food—Agri
11	3.02	0.56%	Hotel—Power
12	2.98	0.55%	Trade—Power
13	2.73	0.51%	Trade
14	2.23	0.41%	Power—Power—Power—Power
15	2.11	0.39%	Agri—Chem—Power
16	1.85	0.34%	Agri—Chem
17	1.67	0.31%	Agri—Agri
18	1.66	0.31%	Hotel
19	1.63	0.30%	Agri—Power—Power
20	1.63	0.30%	Real Est
21	1.55	0.29%	Land T
22	1.36	0.25%	Beverage
23	1.36	0.25%	Edu—Power—Power
24	1.32	0.24%	Water T
25	1.28	0.24%	Public T
SUM	136.14	25.18%	—

SUM in the last row refers to the sum of figures ranking from 1 to 25.

**Table 4:** Top 25 routes of CO_2_ emissions from Urban household Consumption.

Ranks	Data (Mt CO_2_)	Ratio (%)	Routes
0	140.48	9.36	Direct energy use
1	175.54	11.70	Power
2	62.97	4.20	Power—Power
3	22.59	1.50	Power—Power—Power
4	21.30	1.42	Post
5	13.82	0.92	Agri
6	11.29	0.75	Food
7	10.38	0.69	Hotel—Power
8	10.11	0.67	Food—Power
9	9.90	0.66	Food—Agri
10	8.49	0.57	Public T
11	8.22	0.55	Trade—Power
12	8.10	0.54	Power—Power—Power—Power
13	7.53	0.50	Trade
14	7.25	0.48	Edu—Power
15	6.41	0.43	Health—Power
16	5.68	0.38	Hotel
17	5.29	0.35	Agri—Power
18	5.05	0.34	Water—Power
19	4.75	0.32	Post—Power
20	4.47	0.30	Other S—Power
21	3.80	0.25	Real Est
22	3.79	0.25	Food—Agri—Power
23	3.72	0.25	Hotel—Power—Power
24	3.70	0.25	Tran M—FM
25	3.63	0.24	Food—Power—Power
SUM	427.76	28.51	—

SUM in the last row refers to the sum of figures ranking from 1 to 25.

**Table 5:** Top 25 routes of CO_2_ emissions from Government Spending.

Ranks	Data (Mt CO_2_)	Ratio (%)	Routes
1	22.47	4.53	Admin—Power
2	20.99	4.23	Edu—Power
3	11.18	2.25	Admin
4	8.56	1.73	Health—Power
5	8.06	1.63	Admin—Power—Power
6	7.53	1.52	Edu—Power—Power
7	7.45	1.50	Land T
8	5.58	1.13	Edu
9	4.78	0.96	Health—Medi—Power
10	4.56	0.92	Public T
11	4.37	0.88	Admin—Post
12	4.12	0.83	Health
13	3.07	0.62	Health—Power—Power
14	2.95	0.59	Admin—Petro
15	2.89	0.58	Admin—Power—Power—Power
16	2.7	0.54	Edu—Power—Power—Power
17	2.62	0.53	Admin—Hotel—Power
18	2.11	0.43	Admin—Air T
19	1.86	0.38	Other S—Power
20	1.71	0.35	Health—Medi—Power—Power
21	1.7	0.34	Health—Medi
22	1.54	0.31	Admin—Petro—Oil/gas—Power
23	1.44	0.29	Admin—Hotel
24	1.35	0.27	Other S
25	1.23	0.25	Admin—NM
SUM	136.82	27.59	—

**Table 6:** Top 25 routes of CO_2_ emissions from Investment.

Ranks	Data (Mt CO_2_)	Ratio (%)	Routes
1	203.78	7.11	Constr—FM
2	188.34	6.57	Constr—NM
3	74.64	2.60	Constr—NM—Power
4	73.54	2.56	Constr—Power
5	63.5	2.21	Constr—FM—FM
6	57.55	2.01	Constr
7	31.25	1.09	Constr—NM—NM
8	30.89	1.08	Constr—FM—Power
9	28.95	1.01	Speci M—FM
10	26.77	0.93	Constr—NM—Power—Power
11	26.38	0.92	Constr—Power—Power
12	22.2	0.77	Comm M—FM
13	19.79	0.69	Constr—FM—FM—FM
14	19.59	0.68	Constr—Public T
15	17.87	0.62	Tran M—FM
16	16.9	0.59	Constr—FM—FM min—Power
17	16.34	0.57	Speci M—Power
18	13.99	0.49	Constr—Metal—FM
19	13.44	0.47	Constr—Land T
20	12.38	0.43	Constr—NM—NM—Power
21	12.02	0.42	Comm M—Power
22	11.08	0.39	Constr—FM—Power—Power
23	10.74	0.37	Post
24	10.41	0.36	Constr—Chem—Power
25	9.62	0.34	Constr—FM—FM—Power
SUM	1011.96	35.28	—

**Table 7:** Top 25 routes of CO_2_ emissions from Exports.

Ranks	Data (Mt CO_2_)	Ratio (%)	Routes
1	87.51	3.53	FM
2	27.27	1.10	FM—FM
3	22.46	0.91	Metal—FM
4	21.48	0.87	NM
5	20.45	0.82	Chem—Power
6	19.62	0.79	Electro M—Power
7	17.89	0.72	Chem
8	16.85	0.68	Textile—Power
9	16.85	0.68	Water T
10	14.93	0.60	Metal—Power
11	13.26	0.54	FM—Power
12	12.35	0.50	Comm M—FM
13	11.79	0.48	Air T
14	11.54	0.47	Textile
15	10.28	0.41	Electro M—Electro M—Power
16	9.62	0.39	Speci M—FM
17	8.84	0.36	Electri M—NFM—Power
18	8.51	0.34	NM—Power
19	8.5	0.34	FM—FM—FM
20	8.25	0.33	NFM—Power
21	8.16	0.33	Electri M—FM
22	7.33	0.30	Chem—Power—Power
23	7.26	0.29	FM—FM min—Power
24	7.04	0.28	Electro M—Power—Power
25	7	0.28	Metal—FM—FM
SUM	405.04	16.34	—

### Household consumption

**Fig 4:**
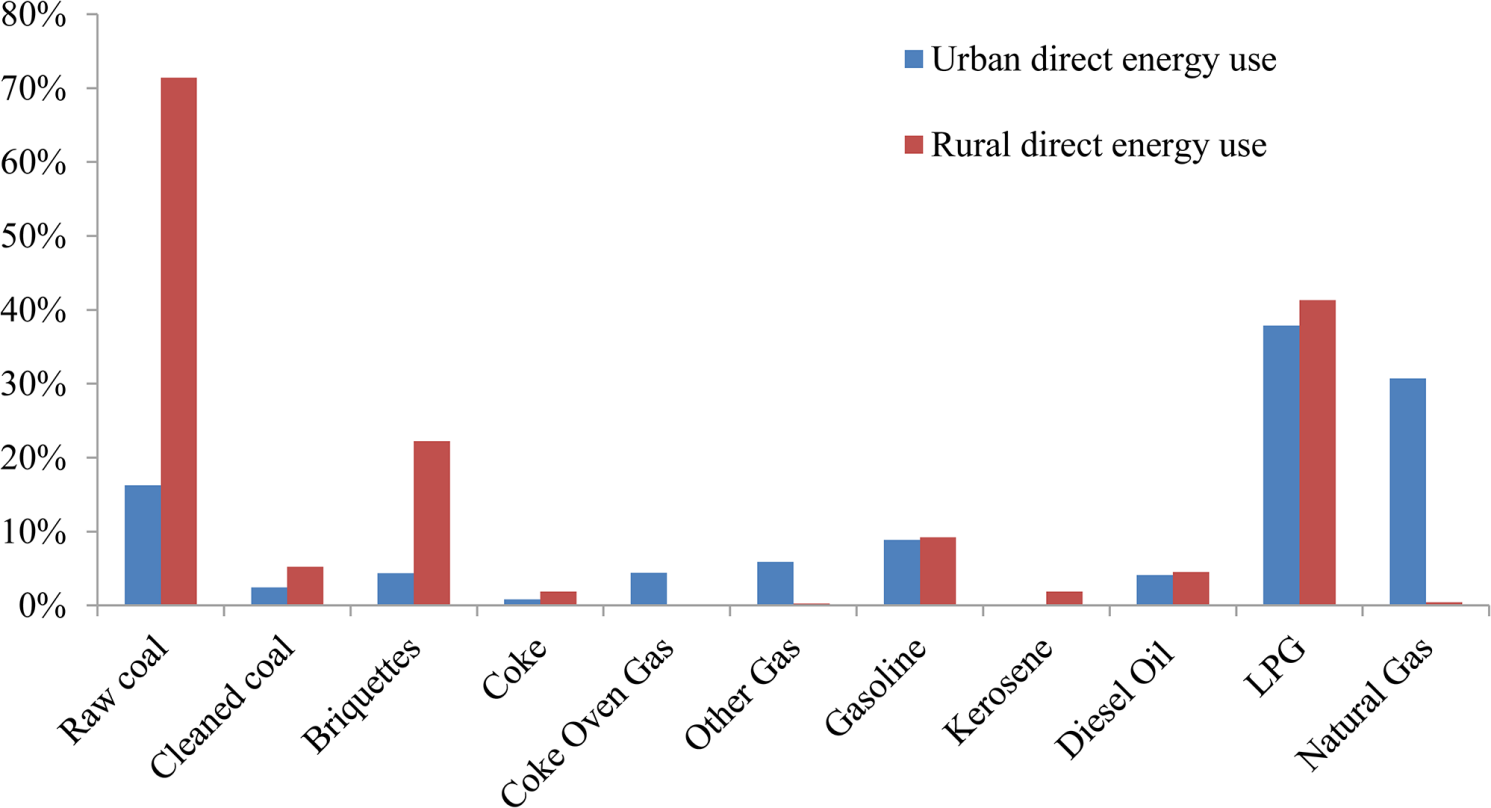
Shares of direct energy use in different forms for rural and urban areas in 2007. Blue and red bars represent ratios for urban and rural areas, respectively. All energy forms are measured in standard quantity (10,000 tons of coal equivalent).

Household emissions from direct energy use (Tables 3 and 4) in rural and urban areas contribute 126.16 Mt and 140.48 Mt CO_2_, respectively. The ratio of direct use emissions to total emissions from household consumption activities in rural area is quite large (23.35%), but the ratio in urban areas is only 9.36%. The reason for this huge difference is the energy use structures in these two areas. As shown in Fig 4, rural areas depend greatly on raw coal and briquettes, whereas urban areas rely heavily on gas fuels. Therefore, mitigation policy towards household direct use emissions should focus on the energy structure in rural areas and raise the share of gas fuels and renewable energy sources, such as solar and biomass.

For the top 25 routes originating from indirect energy use in both rural and urban areas (see Tables 3 and 4), these emissions account for 25%-29% of total emissions from household consumption activities. Many top routes exist as onsite emissions, which have lengths of one and come from direct purchases of final demand (e.g., “Power”, “Agri”, “Post” and “Food”). The largest onsite emissions occur in the demand for electricity and heat (“Power”), which can be ascribed to high dependence on coal for power generation in China. Other onsite emissions lie in food, transportation, agriculture, trade services and real estate sectors, in which urban areas have higher emission values by a factor of 3 to 6 compared to rural areas. Comparisons between top routes in Tables 3 and 4 indicate that chemical sector is rather specific for rural consumption because “Agri-Chem” and “Agri-Chem-Power” only exist in Table 3. Due to our classification, Chem includes seven subsectors (see Table B in S1 File) and Agri includes five subsectors in the original 135 IO table [58]. Focusing on the monetary flows from subsectors in Chem to Agri, we find that chemical fertilizer and chemical pesticides are the most significant. Intermediate flows from the fertilizer sector to the agriculture sector are more than six times greater than those from the pesticide sector. Therefore, CO_2_ emissions from fertilizer and pesticide production in Chem contribute greatly to satisfying rural consumption in Agri. And future mitigation policy should be implemented to limit the unreasonable dependence of agriculture on fertilizer and pesticides and to reduce emission intensities during these chemicals production. As for urban consumption, more emphasis is placed on education, health and other services, as shown in Table 4. Moreover, because the most indirect paths end up in the Power sector, emissions intensity reduction and unreasonable downstream demand cuts in Power can be rather crucial for future mitigation policy. Overall, the results show that future policies should be implemented to change the direct energy use structures in rural areas, to reduce emission intensities and cut unreasonable demand in Power and Chem sectors, and to focus more on urban areas due to its higher magnitudes in the same routes.

### Government Spending

The top 25 routes in Table 5 contribute 27.6% of the total incurred emissions by government spending. These incurred impacts are dominated by various transport modes (e.g., Land T and Public T) and service activities (mainly the Health, Edu and Admin sectors). Indirect routes are significant on the top list and more important than onsite routes. This is common for service sectors because these activities are often not energy intensive (as shown in Fig 1), and they are supported by upstream emission intensive industries. Table 1 further verifies this; the tier 1 ratio is three times the value of tier 0. For example, the Edu sector requires large amounts of power to operate schools and universities (DPI = 18.8) and hospitals in the “Health” sector need both energy and medical products to function well (DPI = 22.75). As for the Admin sector (e.g., administrative authorities at various levels), which is the largest expenditure item in government spending, relevant environmental impacts mainly flow to transport (“Admin-Air T”), power generation (“Admin-Power”), post services (“Admin-Post”) and petroleum products manufacturing (“Admin-Petro”) sectors. To mitigate emissions originating from government spending, future policies should not only manage and supervise onsite energy use in these service sectors but also reduce emission intensities in upstream industries. As for transport sectors, since they are emission intensive, future policies should focus on promoting cleaner fuels (e.g., biomass and hydrogen), energy-saving techniques and publishing relevant transportation standards are also necessary, especially for Land T and Public T sectors.

### Investment

Investment includes both fixed capital investment and inventory change. Fixed capital investment mainly flows to the real estate sector, the industrial sectors and infrastructure construction activities. The magnitude of investment is always greater than the inventory change, and so are the emissions incurred. Top 25 routes contribute to 35.28% of total incurred emissions, as shown in Table 6. Apart from onsite emission routes ranking 6^th^ (“Constr”) and 23^rd^ (“Post”) on the list, all other routes originate from indirect purchases. Most of these indirect emissions stem from the demand for equipment manufacturing (Spec M, 7.75%, Comm M, 6.35% and Trans M, 9.1%) and construction activities (Constr, 57.4%), as shown in the aggregated 56-sector IO table. Often, these demand flow upstream along the production chain and end up in the ferrous metal smelting (FM) and non-metallic smelting (NM) sectors. Although the Power sector is also acting as a crucial end point in indirect routes for investment, it is not as significant as the routes for household consumption and government spending (see Tables 3–5). Combining these investments in the manufacturing and construction sectors with their large DPI values makes it clear that investment contributes a huge share (38%) of total incurred emissions (as shown in Fig 1). Thus, future mitigation policies should focus on direct emission intensity reduction in the major investment sectors and establish standards to regulate their demand for emission intensive upstream materials (such as the demand for steel, glass and concrete in Constr sector), especially for those sectors with large DPI values. This will help encourage emission reductions along the whole production chain.

### Exports

Table 7 shows the top 25 routes resulting from exports, and cumulative emissions are only 16.34% of the total. Compared with other final use elements, this low share indicates that most emissions are dispersed along the production chain. As we can see from Table 7, onsite emissions from the FM, NM, Textile, Water T, Air T and Chem sectors contribute a great deal. Indirect routes, however, mainly start from resource processing sectors (e.g., FM, NM, NFM, Chem and Metal) and equipment manufacturing sectors (e.g., Electri M, Speci M and Electro M and Comm M), and they end up in the two energy intensive sectors: ferrous metal smelting and pressing (FM) and power generation (Power). Additionally, research on embodied technology and quality of manufactured exports in China [69] indicates that low-technology products still dominated the manufactured exports (nearly 53%) in 2007, and shares of medium- (23%) and high-technology (11%) products keep growing, but are still lower than the global averages. Relevant quality results also indicate that most manufactured exports lie in low-quality areas (50.2%) [69].

In summary, exports in 2007 are mainly resource- or emission-intensive metal products, equipment manufacturing and chemical products, in which low technology and low quality prevail. Therefore, future mitigation polices should pay attention to those intensive sectors, try to reduce their direct emission intensities, cut unreasonable demand, and improve the embodied technology and quality in manufactured goods by reshaping exports structures. In other words, future policies should combine environmental guidance with economic structural change.

### Sectoral perspective

After discussing environmental impacts from different final use elements, three representative sectors with a wide variety of business types are selected to illustrate the further implications regarding interrelationships among industries: a large extractive industry (coal mining), a significant secondary industry (construction) and a tertiary industry (public administration). The emissions and ratios of the top 25 routes for specific sectors are shown in Tables 8–11.

**Table 8:** Top 25 routes for unit demand in Coal Mining & Dressing (“Coal”) Sector.

Ranks	Data (g CO_2_)	Ratio (%)	Routes
1	107.55	29.56	Coal
2	59.40	16.32	Coal—Power
3	21.31	5.86	Coal—Power—Power
4	11.24	3.09	Coal—FM
5	10.82	2.97	Coal—Coal
6	7.64	2.10	Coal—Power—Power—Power
7	5.97	1.64	Coal—Coal—Power
8	3.50	0.96	Coal—FM—FM
9	2.74	0.75	Coal—Power—Power—Power—Power
10	2.24	0.62	Coal—Land T
11	2.14	0.59	Coal—Coal—Power—Power
12	1.70	0.47	Coal—FM—Power
13	1.55	0.43	Coal—NM
14	1.51	0.41	Coal—Metal—FM
15	1.28	0.35	Coal—Comm M—FM
16	1.18	0.32	Coal—Petro
17	1.13	0.31	Coal—Coal—FM
18	1.13	0.31	Coal—Speci M—FM
19	1.09	0.30	Coal—FM—FM—FM
20	1.09	0.30	Coal—Coal—Coal
21	1.06	0.29	Coal—Water T
22	1.00	0.28	Coal—Metal—Power
23	0.98	0.27	Coal—Power—Power—Power—Power—Power
24	0.93	0.26	Coal—FM—FM min—Power
25	0.93	0.25	Coal—Rail T
SUM	251.12	69.01	—

**Table 9:** Top 25 routes for unit demand in Construction Sector.

Ranks	Data (g CO_2_)	Ratio (%)	Routes
1	34.76	10.20	Constr—FM
2	32.13	9.43	Constr—NM
3	12.73	3.74	Constr—NM—Power
4	12.54	3.68	Constr—Power
5	10.83	3.18	Constr—FM—FM
6	9.82	2.88	Constr
7	5.33	1.56	Constr—NM—NM
8	5.27	1.55	Constr—FM—Power
9	4.57	1.34	Constr—NM—Power—Power
10	4.5	1.32	Constr—Power—Power
11	3.38	0.99	Constr—FM—FM—FM
12	3.34	0.98	Constr—Public T
13	2.88	0.85	Constr—FM—FM min—Power
14	2.39	0.70	Constr—Metal—FM
15	2.29	0.67	Constr—Land T
16	2.11	0.62	Constr—NM—NM—Power
17	1.89	0.55	Constr—FM—Power—Power
18	1.78	0.52	Constr—Chem—Power
19	1.64	0.48	Constr—FM—FM—Power
20	1.64	0.48	Constr—NM—Power—Power—Power
21	1.61	0.47	Constr—Power—Power—Power
22	1.59	0.47	Constr—Metal—Power
23	1.57	0.46	Constr—Petro
24	1.55	0.46	Constr—Chem
25	1.38	0.41	Constr—Post
SUM	163.52	48	—

**Table 10:** Top 25 routes for unit demand in Public Adminstration Sector.

Ranks	Data (g CO_2_)	Ratio (%)	Routes
1	14.41	11.68	Admin—Power
2	7.17	5.81	Admin
3	5.17	4.19	Admin—Power—Power
4	2.81	2.27	Admin—Post
5	1.89	1.53	Admin—Petro
6	1.85	1.50	Admin—Power—Power—Power
7	1.68	1.36	Admin—Hotel—Power
8	1.36	1.10	Admin—Air T
9	0.99	0.80	Admin—Petro—Oil/gas—Power
10	0.92	0.75	Admin—Hotel
11	0.79	0.64	Admin—NM
12	0.74	0.60	Admin—Petro—Oil/gas
13	0.67	0.54	Admin—Power—Power—Power—Power
14	0.63	0.51	Admin—Edu—Power
15	0.63	0.51	Admin—Post—Power
16	0.6	0.49	Admin—Hotel—Power—Power
17	0.55	0.45	Admin—Land T
18	0.51	0.41	Admin—Rail T
19	0.5	0.41	Admin—Constr—FM
20	0.49	0.40	Admin—Wood—Power
21	0.46	0.38	Admin—Constr—NM
22	0.46	0.38	Admin—Paper
23	0.45	0.37	Admin—Print—Paper
24	0.45	0.36	Admin—Tran M—FM
25	0.38	0.30	Admin—Air T—Petro
SUM	46.56	37.74	—

**Table 11:** Top 25 routes for unit demand in Oil/gas Extraction Sector.

Ranks	Data (g CO_2_)	Ratio (%)	Routes
1	66.59	23.84	Oil/gas—Power
2	50.11	17.94	Oil/gas
3	23.89	8.55	Oil/gas—Power—Power
4	12.33	4.42	Oil/gas—FM
5	8.57	3.07	Oil/gas—Power—Power—Power
6	3.84	1.38	Oil/gas—FM—FM
7	3.16	1.13	Oil/gas—Petro
8	3.07	1.10	Oil/gas—Power—Power—Power—Power
9	1.87	0.67	Oil/gas—FM—Power
10	1.65	0.59	Oil/gas—Petro—Oil/gas—Power
11	1.5	0.54	Oil/gas—Speci M—FM
12	1.41	0.50	Oil/gas—Chem—Power
13	1.3	0.46	Oil/gas—NM
14	1.24	0.44	Oil/gas—Petro—Oil/gas
15	1.23	0.44	Oil/gas—Chem
16	1.2	0.43	Oil/gas—FM—FM—FM
17	1.1	0.39	Oil/gas—Power—Power—Power—Power—Power
18	1.02	0.37	Oil/gas—FM—FM min—Power
19	0.95	0.34	Oil/gas—Comm M—FM
20	0.9	0.32	Oil/gas—Oil/gas—Power
SUM	186.93	66.92	—

### Coal Mining and Dressing Sector

Results show that 363.89 g of accumulated CO_2_ are emitted for every 1 Yuan of final demand in the Coal sector, and the top 25 routes account for 70% of the total incurred emissions (see Table 8). Tracking these routes makes it possible to recognize the interrelationships between Coal and other sectors. Column 1 in Table 8 shows the ranking positions of various routes, and column 2 refers to their incurred CO_2_ emissions (in grams) by unit final demand in Coal sector. For example, route “Coal-Power” indicates that 59.4 g CO_2_ is emitted due to fuel combustion in electricity and heat generation to serve unit final demand for the Coal sector. Column 3 represents ratios of these incurred emissions in each route to total ones. Column 4 shows route details along the production chain.

A 30% share of total incurred emissions comes from onsite energy use, which is rather remarkable; it is almost twice the emissions of the second largest route. This means that the Coal sector contributes a great deal without depending on the upstream industries. Indirect routes indicate that the Coal sector depends greatly on the Power sector, as shown in the routes ranking 2, 3, 6, 9 and 23. It also relies on metal products (e.g., FM and Metal), equipment manufacturing (e.g., Speci M and Comm M) and transport (e.g., Land T and Water T), which can be ascribed to the construction of rigs/equipment and the transportation of coal products. What is more, exports contribute the most in the final demand for Coal sector, and this is quite different from other resource mining industries (FM min, NM min, NFM min, Oil/gas sectors) in which imports dominate.

Therefore, future mitigation policies should focus on energy use management in large domestic coal mining companies, for both fossil fuel combusted onsite and electricity and heat consumed along the production processes. Because raw coal is the major fuel (94.5%) used for generating electricity and heat (see Fig 1) [62], future policies also should focus on rearranging energy structures, limiting raw coal consumption and promoting other cleaner or renewable energy forms. Moreover, attention should be directed to resources and energy safety issues, especially for crude oil, gas, metal ores (e.g., FM min and NFM min) due to their huge imports in final demand. As for the huge exports in Coal sector, future policy should analyze benefits and costs considering the energy and environmental dilemmas in China.

### Construction Sector

Because investment flows into infrastructure development and real estate market have soared in recent years [68], the construction sector requires special focus. Similar to the Coal sector, the top 25 routes in the Constr (see Table 9) sector account for half of total incurred emissions (48%), and the sectoral unit final demand can stimulate 340.9 g CO_2_. The results show that onsite emissions are less significant (merely 2.88% of the total) and a majority of large emission routes originate from indirect purchases (such as “Constr-NM” and “Constr-Power”) flowing upstream to NM, FM and Power sectors. Other intensive sectors, such as chemical products (Chem), refinery products (Petro), metals (Metal) and transport services (Land T, Public T and Post) are also strongly required. Unlike the Coal sector, which belongs to upstream industry and has only a small DPI value (0.14), the downstream Constr sector has a great demand pulling effect (DPI value equals 33.2) on the total economy. Additionally, final demand for Constr mainly resides in fixed capital formation and barely shows in imports, as seen in the aggregated IO table, indicating that incurred emissions in this sector are mostly for domestic use. Therefore, future mitigation work should pay great attention to Constr and similar sectors that account for large shares of domestic emissions, especially those with large DPI values. Relevant sectoral policies should also focus on managing onsite energy use, promoting new building technology, cutting unreasonable construction plans and setting up new standards for upstream materials as declared in the “Investment” section above. Considering the booming real estate markets and infrastructure development in China and their huge demand pulling effects on emission intensive upstream industries through Constr sector, future development policies in these two fields must combine economic concerns with environmental ones to achieve sustainable development.

### Public Administration Sector

Unit final demand in the Admin sector can stimulate 123.4 g CO_2_ along the production chain, and the top 25 routes account for 37.7% of the total incurred emissions (see Table 10). The largest incurred impacts mainly come from the indirect purchase route (“Admin—Power”), which contributes 11.68% of total emissions, whereas onsite emissions contribute 5.8%. Various tertiary sectors (e.g., Post, Transport, and Hotel) and secondary sectors (e.g., Constr, Petro and NM) are strongly required in the top 25 list. The DPI value for the public administration sector is 17, which is similar to other large service sectors, such as Edu (DPI = 16.8) and Health (DPI = 22.75). Moreover, final use in Admin sector mainly consists of government spending and is the largest cell in it, as shown in the original IO table. All of these facts remind us that future policies must not ignore service sectors due to their high pulling effects on emissions and the economy. Future policies should not only manage and supervise onsite energy use in these service sectors but also promote reduction of emission intensities upstream from a systematic viewpoint.

Additionally, we compare the above results with two other SPA studies from Peters and Hertwich [25] and Huang et al. [23] in both the final use and sectoral perspectives. In the final use viewpoint, our results resemble those tier distribution results in household consumption and government spending for Norway [25], but they are different from those results in exports. This difference can be explained by direct emissions from inter-national shipping and direct processing in the oil and gas sector, which are unique for Norway. As for incurred emission routes from each final use elements (household consumption, government spending and exports) [25], the differences are clear. China often ends up in emission intensive electricity and heat (Power) sector while Norway relies on transport sectors a lot, which can be ascribed to their very different energy structures. The Norwegian economy has low emission intensity due primarily to the use of hydropower in electricity generation, whereas the Chinese economy depends greatly on coal. Another significant difference lies in the export sector; Norway is dominated by international shipping and oil/gas production (over 40%) due to its resources, but China shows a distributing nature in many small routes for industrial products (e.g., metal, equipment manufacturing and chemicals). Additionally, routes in the final use viewpoint have much in common for the same economic reasons, such as high demand for Food and Agri products in household consumption and large requirements for Health, Edu and Admin services in government spending.

In the sectoral viewpoint, the Oil and gas extraction sector (Oil/gas) is chosen for demonstration (see Table 11). Unlike the environmental impacts used in this study, the impact indicator in Huang et al. [23] is GHG equivalents (CO_2_-eq), which includes CO_2_, CH_4_ and other GHGs. These incurred CO_2_-eq routes from unit sectoral demand in Australia and US are computed using SRIO and SPA based on 2001 and 2002 IO table, respectively [23]. The results reveal that 67%, 89% and 93% of emissions come from the top 20 routes for China, Australia and the US, respectively. The onsite emissions, which might occur directly at rig/drilling sites in the US and Australia, are quite high (85.2% and 80.9%), and China contributes just 18%. However, indirect emissions from the Power sector dominates the top ranks (nearly 24%) in China and is more significant than in the US (3%) and Australia (8%), which might be due to China’s high dependence on coal for electricity and heat. These huge differences might be due to different energy use patterns in the Oil/gas sector. Large demand for petroleum products, ferrous metals, equipment and machinery are all shown in top 20 routes of three target studies. In summary, our SPA results in two viewpoints show both common results to earlier studies and special characteristics due to China’s own economic and energy structures. Therefore, it can be seen that SPA is a useful tool for analyzing the sectoral interactions among industries and can reflect real circumstances. Because China has been the world’s largest emitter, it is practical to extract crucial emission routes along its production chain and to use these empirical results as guides for developing reasonable mitigation polices.

## Conclusions

Theoretically, this paper is different from previous studies that use SRIO and SPA in three ways. First, our model is based on the original 135-sector competitive IO table with error items, industrial energy consumption data and an energy balance sheet for 2007. Sectors are aggregated into 56 categories to balance economic and energy details by considering data availability and our research purpose. Second, the regional technical coefficients matrix is selected to reflect the actual technical interrelationships among sectors in China. Although the relevant competitive IO system cannot display enough imports details, it is still a reasonable substitute for the MRIO model and can meet our research demands. To derive the actual domestic final use, imports and exports in bonded warehouses and zones and three other importing sub-groups are removed, and the remaining importing elements in final use (C_2_, I_2_, G_2_) are eliminated based on customs data. Third, this imports-adjusted IO table is used to implement empirical SPA studies in both the final use and sectoral perspectives.

The results indicate that future mitigation policies on household consumption should be implemented to change the direct energy use structures in rural areas, to reduce emission intensities and cut unreasonable demand in Power and Chem sector, and to focus more on urban areas due to its higher magnitudes than rural areas in the structural routes. Impacts originating from government spending should be tackled by managing onsite energy use in 3 major services sectors (Edu, Health and Admin) and by promoting cleaner fuels (e.g., biomass and hydrogen) and energy-saving techniques in the Transport sector. As for investment, special attention should be paid to the major manufacturing and construction sector with large DPI values, and standards should be established to regulate the upstream material demand and push emissions down along the production chain. Apart from the similar methods above, mitigating policies in exports should also consider improving embodied technology and quality in manufactured products to achieve sustainable development. Detailed sectoral analyses in Coal indicate that future mitigation policies should concentrate on onsite energy use in large domestic coal mining companies, rearrange energy consumption structure by substituting coal shares, and call for attention in resources (e.g., metal ores) and energy (e.g., oil, gas and coal) safety issues due to their dominating imports or exports. Additionally, results in Admin and Constr sectors show that major secondary and tertiary sectors with large DPI values should not only cut their direct emission intensities but also be planned together with upstream industries in an integrated way due to their high demand pulling effects.

In summary, this paper constructs an adjusted SRIO framework by considering final use elements produced domestically, and it uses SPA technique to highlight crucial routes stimulated by these elements along the production chain in both final use and sectoral perspectives. This work is the first detailed empirical analysis of the Chinese economy using SPA, and it shows detailed mitigation strategies and suggestions, which demonstrates that SPA is a useful tool for empirical studies. Future studies of China using SPA can extend to time series analysis by comparing changes in different years, combine SPA with other methods such as structural decomposition for deeper analysis (e.g., SPD), or implement empirical studies based on detailed multi-regional input-output models (MRIO).

## Supporting Information

S1 File Supporting Information. Sector classification in this study **(Table A)**. Mapping of 135 sectors in Original 2007 Input-Output table to 56 sectors in this study **(Table B)**. Mapping of 44 sectors in the Energy balance sheet and Industrial Energy consumption sheet to the 56 sectors in this study **(Table C)**. Two different separation methods for sectors 1–37: simple proportional separation method and a method based on the customs statistics **(Table D)**. Separation ratios based on results in Table D **(Figure A)**.
(XLSX)
